# Eating Together, Eating Alone: A Cross-Sectional Survey of Associations Between Social Eating Contexts, Mealtime Emotions, Technology Use, and Loneliness in UK University Students

**DOI:** 10.3390/ijerph23060739

**Published:** 2026-06-01

**Authors:** Laura Chandler, Yanyan Li, Diya Agarwal, Jan Antkiewicz, Judah Chike-Michael, Domenico Giacco, Sagar Jilka, Daniel Mensah, Ian Saunders, Carla Toro, Helena Tuomainen

**Affiliations:** 1 Warwick Medical School, University of Warwick, Coventry CV4 7AL, UK; laura.a.chandler@warwick.ac.uk (L.C.); yanyan.li.1@warwick.ac.uk (Y.L.); jan.antkiewicz@warwick.ac.uk (J.A.); domenico.giacco@warwick.ac.uk (D.G.); sagar.jilka@warwick.ac.uk (S.J.); carla.toro@warwick.ac.uk (C.T.); 2 Centre for Evidence and Implementation Science, School of Social Policy and Society, University of Birmingham, Birmingham B15 2TT, UK; d.mensah@bham.ac.uk; 3 Department of Computer Science, University of Warwick, Coventry CV4 7AL, UK; ian.saunders@warwick.ac.uk; 4 School of Nursing and Midwifery, University of Worcester, Worcester WR2 6AJ, UK

**Keywords:** loneliness, university students, social eating, student wellbeing, digital technology

## Abstract

**Highlights:**

**Public health relevance—How does this work relate to a public health issue?**

- Loneliness is a growing public health concern amongst university students, linked to poorer mental health outcomes.
- This study examined how social eating habits and practices and mealtime experiences relate to loneliness in a higher education setting.

**Public health significance—Why is this work of significance to public health?**

- Findings identified that specific mealtime habits and practices (e.g., eating alone, technology use) are associated with higher loneliness.
- The study identified first-year students as a potentially higher-risk group for loneliness within university populations.

**Public health implications—What are the key implications or messages for practitioners, policy makers and/or researchers in public health?**

- Interventions should promote inclusive and accessible opportunities for eating together with others to help reduce loneliness amongst students.
- Universities should consider designing dining environments that reduce anxiety and stigma associated with both eating alone and eating with others.

**Abstract:**

**Background**: Loneliness is prevalent amongst university students and may be influenced by social eating behaviours. This study explored associations between loneliness and social eating habits and practices and examined whether loneliness varies by demographic characteristics and mealtime behaviours. **Methods**: A cross-sectional online survey was conducted amongst 255 undergraduate and postgraduate students at a UK campus-based university. Loneliness was measured using the UCLA Loneliness Scale (ULS-8). Self-reported social eating habits, emotional experiences during mealtimes, and technology use while eating were assessed. **Results**: The mean loneliness score was 18.27 (SD = 4.90), with 16.1% of participants experiencing severe loneliness. Loneliness did not differ across most demographic groups, except by year of study, with first-year undergraduates reporting higher loneliness than PhD students. Higher loneliness was reported by students who felt embarrassed or lonely when eating alone, were apprehensive about eating with others, or lacked someone to eat with. Greater use of electronic devices or television during meals was also associated with higher loneliness. **Conclusions**: Loneliness is common amongst university students and is associated with social eating habits/practices and emotional experiences during mealtimes. Interventions promoting social eating could address discomfort and anxiety related to eating alone or with others.

## 1. Introduction

Loneliness is a distressing feeling associated with having fewer or lower-quality social relationships than desired [1]. It can be triggered and maintained by both fixed (e.g., demographic) and modifiable (e.g., socioenvironmental) factors [2]. The transition to university often involves separation from close relationships, adapting to new environments, and establishing new social networks and support systems. Building new friendships is a key challenge during this period [3], and university can trigger both short- and long-term loneliness [2], with nearly one-in-four university students feeling lonely ‘all’ or ‘most’ of the time [4]. Loneliness is particularly pronounced amongst disabled students, those from minoritised ethnic or gender groups, students living at home or with caring responsibilities [5], and international students [6]. The COVID-19 pandemic intensified this issue, with strict social distancing measures doubling loneliness prevalence amongst those aged 18–25 across the EU [7]. The pandemic also magnified the associated risks of depression, anxiety, stress, suicidal ideation [8,9,10], and harmful behaviours, such as alcohol, drug misuse or internet addiction [11]. Loneliness amongst university students is therefore a significant public health concern and more research should explore factors that can intensify or alleviate feelings of loneliness in this population group.

Given the impact of loneliness on student wellbeing, exploring everyday practices that foster social connections, such as shared meals, may offer promising avenues to alleviate loneliness. Commensality is defined as the practice of eating with others at the same table [12]. Sharing meals symbolises community and provides opportunities for social interaction, information exchange, and the development of supportive relationships [13,14]. Through these interactions, commensality can support social bonding by creating a sense of belonging, validating cultural identity and strengthening wellbeing [15,16]. Research has demonstrated that individuals who regularly engage in communal eating report greater happiness, higher life satisfaction, increased social engagement, and more dependable friendships [13,15]. However, commensality can also present disadvantages such as conflicts during mealtime conversations or discomfort in shared eating settings [17].

Opposite to commensality is eating alone or solo dining. Eating alone, especially in public, has been associated with negative feelings such as loneliness, unhappiness and depression in countries such as the UK and Thailand [18,19,20], often perceived as a sign of social isolation. Women, in particular, report higher levels of loneliness when eating alone and are at increased risk of disordered eating behaviours in the absence of commensal dining [21,22]. Although solo dining amongst adult populations can be associated with loneliness, higher depression and poorer nutrition [23,24], it can also be viewed more positively, such as being associated with freedom, self-discipline, efficiency or independence, depending on cultural contexts and perspectives [25,26]. The findings from cross-cultural studies suggest that the meaning of eating alone is culturally embedded, reflecting differing social norms around independence, emotions, and everyday routines.

At university, developing new commensal circles, i.e., networks of people who eat together [27], is particularly important for students adjusting to a new social environment [19]. Academic demands, busy schedules, and conflicting timetables often disrupt social eating, which can lead to meal skipping or eating alone [28]. Although solo eating and reliance on fast food is often convenient, many students express a preference for communal meals, valuing the social interaction and the opportunity to eat more varied and often healthier foods [28,29]. Such practices are associated with greater perceived social support, which is strongly linked to health, wellbeing, and academic success [21], and could, in turn, help alleviate loneliness.

The term “social eating habits and practices” encompasses here both commensality and eating alone, i.e., whether one eats with others or alone, including in the presence of media such as television or smartphones, as well as other social aspects associated with eating occasions, such as their timing, context, location, and the feelings related to eating alone or with others [15,16,19]. While many aspects of eating behaviour become habitual or automatic through repetition (e.g., routinely eating dinner in front of the television after work), the social dimension of eating often involves conscious intention, coordination, or awareness, such as arranging a meal with friends or choosing to avoid eating alone, and can therefore be understood as a practice [30].

Most studies on the relationship between social eating habits/practices and mental health have focused on elderly or paediatric populations [31,32], with relatively little research examining the impact of eating alone or commensality amongst young adult populations. In the UK, a qualitative study examined changes in university students’ eating practices, commensality, and loneliness before and during the COVID-19 lockdown [19]. However, quantitative research examining the relationship between students’ social eating habits/practices and loneliness in UK university settings remains limited, particularly during transitional stages like starting university when supportive social eating networks are being established [33]. As part of a wider intervention development project linked to loneliness and social eating practices amongst university students, this study addresses this gap by examining how social eating habits and practices relate to loneliness amongst UK university students.

### Aims and Research Questions

This study explored levels of loneliness amongst UK university students and examined the relationship with social eating habits and practices. The research questions for this study were:

1. What are the levels of loneliness reported by university students in this study?
2. Which demographic groups of university students report higher levels of loneliness in this study?
3. What is the relationship between social eating habits and practices and reported loneliness amongst university students in this study?

## 2. Materials and Methods

### 2.1. Study Design

The study consisted of a cross-sectional survey, which was administered online via Qualtrics survey software (Qualtrics, Provo, UT, USA; 2024-2025). Ethical approval for the study was granted by the Biomedical and Scientific Research Ethics Committee (BSREC) at the University of Warwick, UK (REF: BSREC 79/23-24 AM01).

### 2.2. Participants

University students, both undergraduate and postgraduate, enrolled at a single university in the West Midlands, UK, were eligible to participate in the study. There were no other specific inclusion or exclusion criteria. Participants were recruited using convenience sampling. No predetermined sample size was set. Instead, all available and willing participants during the recruitment period were included.

### 2.3. Procedure

An email invite and poster, which included both a link and QR code to the survey on Qualtrics, were disseminated across the university via several channels. These included departmental communications, wellbeing services, the student union, campus eating venues and university social media pages. The survey was open for participation between November 2024 and February 2025.

On accessing the survey, participants were presented with an online participant information sheet, which outlined details of the study, contact details for the research team and information on relevant support services. Participants were then asked to complete an online consent form before proceeding with the survey. Once consent was provided, participants completed the survey, which took approximately 10–15 min. Following completion of the survey, participants had the option to enter a prize draw for a chance to win a £50 shopping voucher. A link was provided at the end of the survey, which redirected participants to a separate Qualtrics page where they could enter their email address for the purpose of the prize draw.

### 2.4. Measures

Participants were asked about their demographic characteristics including age, gender, ethnicity, country of origin, length of living in the UK, religion, current living situation, number of people in household, year of study and university faculty.

#### 2.4.1. Loneliness

Loneliness was measured using the UCLA loneliness scale (ULS-8; [34]). The measure consists of eight statements (6 negatively worded, 2 positively worded), related to feelings of loneliness and social connections with others. Responses are on a scale from ‘Never’ (1) to ‘Always’ (4). The total score ranges from 8 to 32, where higher scores indicate higher levels of loneliness. The measure has a high level of validity and reliability in university student samples [35,36].

#### 2.4.2. Social Eating Habits and Practices

Social eating habits and practices were defined as patterns of eating behaviour that reflect whether individuals eat alone or with others, the contexts and locations in which eating occurs, feelings or emotional experiences during mealtimes, and the use of digital technology while eating.

Measures of social eating habits and practices were developed specifically for this study to capture key aspects of student eating practices relevant to university life. Item development was informed by existing research on commensality, solo dining, and loneliness, and was further refined through input from student researchers as part of a Patient and Public Involvement (PPI) process. This ensured that the measures reflected lived student experiences and were relevant to the university context.

The final survey included multiple domains of social eating habits and practices: (1) meal patterns (e.g., whether participants ate breakfast, lunch, snacks, and dinner alone or with others during a typical term-time period), (2) eating locations (e.g., home, on-campus venues, and off-campus settings), (3) reasons for eating alone, (4) emotional experiences when eating alone or when eating with non-close peers in university settings, and (5) use of digital technology during mealtimes (including frequency of device use, television viewing, and perceived use of digital platforms for social connection).

Response formats varied by item type and included dichotomous, categorical, and Likert-style scales. Full item wording, response options, and coding procedures are provided in the Supplementary Materials.

As these measures were study-specific and designed for exploratory purposes, formal psychometric validation was not conducted. However, they were grounded in established theoretical and empirical work and refined through PPI input to enhance face validity and relevance to the target population.

### 2.5. Data Analysis

Participant characteristics were summarised through descriptive statistics to gather an overall picture of the sample, with numbers and percentages reported for each of the demographic characteristics. Descriptive statistics were also performed to gauge the levels of loneliness (RQ1) from the total score on the ULS-8.

Loneliness levels of different demographic groups were then explored (RQ2), using independent t-tests (for binary variables) or one-way ANOVAs (for variables with three or more categories) to examine the difference in reported loneliness between different demographic groups.

Exploratory analyses using independent t-tests or one-way ANOVAs were then performed to examine the differences between social eating habits/practices and reported loneliness of university students (RQ3). Specific analyses included examining the difference in loneliness between participants who selected they eat alone for certain meals, as well as alone at certain venues, compared with those who did not select they eat alone. Analyses also examined differences in loneliness in participants’ reported reasons for eating alone, differences in feelings experienced when eating alone and feelings experienced when eating with others. Differences in participants’ loneliness on their digital technology use during mealtimes was also examined.

Where significant differences were found from ANOVAs performed to answer RQ2 and RQ3, Tukey HSD post hoc tests were performed to identify differences between specific groups.

Additionally, 95% confidence intervals (CI) are reported for Cohen’s d and η^2^ where appropriate (standardised effect sizes).

Participants were not required to answer every question, resulting in item-level missing data. Consequently, sample sizes vary across demographic variables and reported social eating habits/practices for these descriptive statistics. The exact numbers for each variable are reported in relevant tables. For inferential analyses involving loneliness, listwise deletion was used (complete-case analysis): only participants who completed the loneliness questionnaire and the relevant demographic or social eating habits/practices variable were included. As a result, all analyses including loneliness are based on the same subset of participants (n = 255).

Data analysis was performed in SPSS Version 29 [37].

## 3. Results

### 3.1. Participant Characteristics

**Figure 1:**
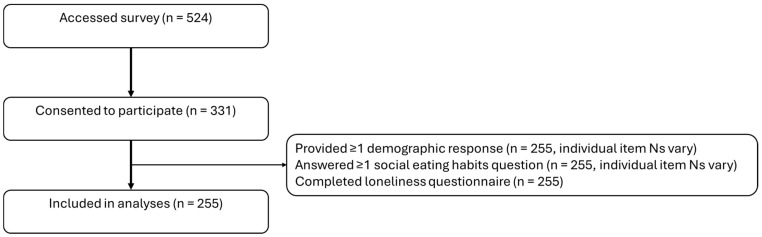
Participant flow through the survey.

A total of 524 individuals accessed the survey on Qualtrics. There were 193 respondents who did not proceed after the information sheet and consent form. An additional 73 participants started but did not complete the survey, with a total of 258 participants completing the survey. Three of these participants did not meet the eligibility criteria for the study and were subsequently removed. A total of 255 participants were included in the analyses (see Figure 1).

The majority of participants were in the 18–20 years age group (49.8%), identified as female (75.8%), of White ethnicity (61.8%), and were from the UK (70.9%). Most participants reported having no religion (56.4%), were living with other students in private accommodation (39.9%), were undergraduate first-year students (27.1%) and were from the Faculty of Science, Engineering and Medicine (55.0%). See Table 1 for further details.

### 3.2. Loneliness Amongst University Students

#### 3.2.1. Levels of Loneliness

The average loneliness score for participants on the ULS-8 was 18.27 (SD = 4.897), indicating that participants were experiencing some degree of loneliness (Table 2). Although there is no standard cut-off score for the ULS-8, previous research has highlighted that a score of 24 could be a potential cut-off for considering severe loneliness [38]. In this study, 16.1% of participants scored above the cut-off, indicating severe levels of loneliness (Table 2).

#### 3.2.2. Differences in Loneliness of Different Demographic Groups

There was a significant difference in students’ living situation (*F* (6, 245) = 3.163, *p* = 0.005, η^2^ = 0.072, CI: 0.01, 0.12); however, post hoc tests via Tukey HSD did not identify any significant difference between specific groups (*p* > 0.05). The number of people living in a household was not significant (*F* (4, 201) = 1.971, *p* = 0.100, η^2^ = 0.038, CI: 0.00, 0.08).

A significant difference was found in participants’ year of study (*F* (6, 243) = 2.786, *p* = 0.012, η^2^ = 0.064, CI: 0.00, 0.11). Post hoc tests via Tukey HSD identified significant differences between undergraduate first-year students and postgraduate PhD students (of any year of PhD study) (*p* = 0.018, CI: 0.34, 6.29), with undergraduate first-year students reporting significantly higher loneliness levels than Postgraduate PhD students (mean diff. = 3.313, SE = 1.001). There were no other significant differences found between loneliness and the other demographic variables (all *p* > 0.05).

#### 3.2.3. Differences in Loneliness in Relation to Social Eating Habits and Practices of University Students

Several aspects of social eating habits/practices were associated with loneliness, including mealtime context, emotional experiences, and technology use (see Table 3).

Meals eaten alone. Eating alone at most mealtimes, including morning snack, lunch, afternoon snack, and dinner, was associated with higher loneliness compared with not eating alone at these times (all *p* < 0.05). The largest difference was observed for dinner. No significant association was found for eating breakfast alone.

Venues eaten at alone. Eating alone at home, on campus (e.g., cafés), and in off-campus venues was associated with higher loneliness (all *p* < 0.05). Eating alone outdoors on campus was not significantly associated with loneliness.

Reasons for eating alone. Loneliness differed significantly according to reported reasons for eating alone (*p* = 0.001). Students reporting social barriers such as apprehension about reaching out to others, discomfort eating with others, or lacking someone to eat with, experienced higher loneliness compared with those who did not eat alone (Table 4).

Feelings experienced eating alone at a university venue. Emotional experiences while eating alone were significantly associated with loneliness (*p* < 0.001). Students reporting negative emotions (e.g., embarrassment, discomfort) experienced higher loneliness, whereas those reporting positive emotions (e.g., enjoyment, relaxation) experienced lower loneliness.

Feelings experienced eating with others (not close friends) at a university venue. A similar pattern was observed when eating with other students (not close friends) (*p* < 0.001). Students reporting negative emotions, such as discomfort or embarrassment, experienced higher loneliness, while those reporting positive emotions, such as being happy or carefree, experienced lower loneliness.

Use of digital technology during mealtimes. More frequent use of electronic devices and watching television during mealtimes were associated with higher loneliness (*p* < 0.05), with the highest loneliness observed amongst students reporting constant use. The type of platform used (*p* = 0.927) and students’ attitudes toward using digital technology to connect with others during meals showed no significant association (*p* = 0.720).

## 4. Discussion

### 4.1. Summary of Key Findings

This study explored loneliness levels amongst university students and examined associations between loneliness and social eating habits and practices. The sample indicated some degree of loneliness on average, with 16.1% experiencing severe loneliness. Demographics were generally not associated with differences in loneliness, except for year of study, with first-year undergraduate students reporting significantly higher loneliness than PhD students.

Loneliness was significantly associated with various social eating patterns. More students ate alone than with others at lunch and afternoon snack, which are times when students are typically on campus. Students who reported eating alone during snacks, lunch, or dinner experienced significantly higher loneliness than those who did not report eating alone. Students who felt apprehensive about reaching out to others or lacked eating companions had significantly higher loneliness levels.

Furthermore, students who experienced negative emotions when eating alone or with other students at university (but not close friends), such as being embarrassed, ashamed, or lonely, reported significantly higher loneliness levels compared with those who felt happy, relaxed, or carefree. Finally, frequent use of digital technologies during mealtimes, including small electronic devices or watching TV, was associated with higher loneliness.

#### 4.1.1. Loneliness Varies by Year of Study

The findings related to loneliness align with previous research highlighting high levels of loneliness amongst students, including post-COVID increases [39,40], and impacts on wellbeing [41,42]. Loneliness levels appear to have remained elevated compared with pre-pandemic levels, when only 3.2% reported severe loneliness [43]. First-year undergraduate students had significantly higher loneliness scores than PhD students, suggesting the transition to university is a particularly vulnerable period [44]. Unlike postgraduate students, who typically have established social networks including partners and long-term friendships [41,45], many undergraduate students experience the move to university as their first time leaving home and separating from school friends. This transition, coupled with the pressure to make new friends, may contribute to loneliness and isolation [44]. In the UK, this vulnerability may be compounded by housing arrangements: first-year students are normally housed in halls of residence without the ability to choose housemates, whereas in subsequent years students typically group together as friends to rent from the private market, which may explain reduced loneliness over time [46].

#### 4.1.2. Timing and Setting of Mealtimes Matter

Most students reported eating breakfast alone; however, this was not associated with loneliness. This may reflect a broader social norm and established habit in the UK and elsewhere, including the US, where breakfast is commonly eaten alone [47,48,49,50]. In relation to young people, breakfast is also the meal most frequently skipped [51] and the least likely to be shared with family members [52,53]. Young adults in the UK and other European countries tend to socialise over meals later in the day rather than breakfast [54], a pattern that appears to emerge during adolescence [50]. In contrast, a cross-sectional study found that university students in Korea were less likely to eat breakfast alone than lunch or dinner [55], suggesting potential cultural differences in meal-related social practices and the social significance attached to breakfast.

The new university environment requires students to make decisions about where and with whom to eat. Over half of students eating lunch in our sample ate it alone, and the setting in which students ate alone influenced loneliness. Eating alone in university-owned venues or cafés, or in off-campus venues, was consistently linked with increased loneliness. However, eating alone outside on campus (e.g., in green space or at pop-up food trucks) was not associated with increased loneliness, suggesting that outdoor settings may lessen the social salience of solitary eating and make it feel less socially exposing than eating alone in enclosed dining environments. Alternatively, this pattern may reflect the broader benefits of exposure to green space for reducing loneliness and supporting mental wellbeing [56,57].

Solo dining in restaurants is becoming increasingly socially acceptable [58]; however, eating alone in university dining venues may be particularly challenging for students, especially when surrounded by groups of peers eating together. In the present study, many students associated solitary eating in these settings with feelings of embarrassment and discomfort. Similarly, a qualitative study conducted in the UK found that eating alone in university venues was associated with negative emotions and perceptions amongst most, although not all, participants [19]. While a few graduate students reported feeling comfortable eating alone when surrounded by fellow students—viewing it as an opportunity for personal time—other students described discomfort and a tendency to rush through their meals [19]. This may reflect a broader fear of social evaluation, whereby solitary eating is interpreted negatively by others, potentially reinforcing feelings of loneliness and exclusion. More broadly, university eating cultures often appear to privilege shared meals, with eating alone in public dining spaces carrying a degree of perceived stigma and signalling social disconnection from peers [25,59]. Such perceptions may be amplified by the strong social expectations associated with university life, particularly the expectation that students should form new friendships and social networks during this period [60].

Eating alone at home (student accommodation) was also associated with increased loneliness. The UK qualitative study found that living in shared student accommodation did not necessarily lead to communal eating practices; instead, many students ate separately and at different times [19]. Eating alone at home may feel particularly isolating for new students, as physical proximity in shared accommodation does not necessarily translate into social connection, and the absence of shared mealtimes may heighten awareness of being alone. This may be especially pronounced amongst students accustomed to eating with family members prior to university, given the evidence that regular family meals have a protective effect on the mental health and wellbeing of young people [61,62].

#### 4.1.3. Role of Digital Technology

In situations where students find themselves eating alone, turning to digital technologies such as television, streaming services, and mobile phones for comfort and companionship is perhaps unsurprising, and has also been identified in the previous UK qualitative study [19]. Given the amount of time young people spend on digital devices, it is notable that students reporting always having digital company while eating—whether on campus or at home—experienced the highest levels of loneliness. Eye-tracking studies suggest that highly lonely young adults may develop habitual avoidance of socially threatening stimuli [63,64]. Digital technologies may facilitate this avoidance by distracting individuals from uncomfortable feelings associated with loneliness. Although digital technologies can also help individuals feel connected to others during mealtimes [19,65], they may also reduce opportunities for in-person social interaction, potentially reinforcing solitary eating behaviours.

### 4.2. Limitations

While the study provides valuable insights into the loneliness and social eating habits and practices of university students, several limitations should be acknowledged. First, due to the cross-sectional design of the survey, the study cannot determine causality between social eating habits/practices and loneliness. Although associations were identified, the direction of these effects remains unclear. Students who are already lonely may be more likely to eat alone, yet loneliness may also lead to heightened discomfort in social situations over time, including shared mealtimes.

Second, the measure of social eating habits and practices did not capture the frequency of eating alone or the specific nature of eating companions (e.g., friends, family members, romantic partners, or university peers). As such, the findings cannot distinguish whether different types or frequencies of social eating are differentially associated with loneliness. Future research should incorporate more detailed measures of social eating contexts and interpersonal relationships during meals.

Third, recruitment was via convenience sampling. Students with a pre-existing interest in loneliness, social habits of students or general student wellbeing, may have been more likely to participate, possibly increasing the risk of self-selection bias. However, convenience sampling may still provide relatively unbiased estimates of the association between social eating habits/practices and loneliness, if the relationship between these variables is not systematically distorted by who chooses to participate [66]. Nevertheless, the sample may not accurately reflect levels of loneliness in the wider student population.

Fourth, participants were recruited from a single university, and the sample consisted predominantly of female participants and participants of White ethnicity, limiting the generalisability of the findings. Institutional differences, as well as variations across demographic backgrounds, may impact both experiences of loneliness and social eating habits and practices. Furthermore, relative to the total number of students enrolled at the university, the sample size was small, which may further limit the generalisability of the findings.

Although sample sizes differed across groups, with some groups containing relatively few participants, the assumptions of ANOVA (normality and homogeneity of variances) were met. Unequal group sizes are therefore unlikely to have substantially influenced the results. However, future studies with larger samples should aim for more balanced group sizes to further strengthen statistical power. Finally, although the loneliness measure used was a validated tool, the use of self-report measures increases the risk of social desirability bias.

### 4.3. Implications for Practice and Future Research

Reducing loneliness amongst university students has been identified as a key intervention target [67,68], given rising levels of student loneliness and poor mental health, alongside increasingly over-stretched university wellbeing services. Facilitating shared mealtimes may represent a promising strategy, given the association between eating alone and loneliness, especially in public spaces. Targeted initiatives promoting commensality could therefore be especially beneficial for students at greater risk of loneliness, including first-year undergraduate students. Features that reduce the stigma of eating alone and create opportunities for low-pressure, positive social interactions may further increase the uptake and effectiveness of such initiatives. However, the present findings suggest that simply increasing opportunities for shared eating may be insufficient. Students who reported social barriers to eating with others, such as apprehension about reaching out, discomfort eating with others, or lacking someone to eat with, experienced particularly high levels of loneliness. Future interventions should therefore address not only the practical opportunities for commensality, but also the social anxieties and interpersonal barriers that may prevent students from engaging in shared mealtime experiences.

Digital commensality tools have emerged following the COVID-19 pandemic as innovative approaches to reducing social isolation and loneliness [65,69]. However, given the association between digital technology use during meals and higher levels of loneliness, such tools should be developed and implemented cautiously. In particular, it is important that they complement rather than replace opportunities for in-person social interaction without inadvertently reinforcing solitary eating behaviours.

Further research should examine social eating habits and practices of students and loneliness longitudinally to identify causal relationships over time. Future research should also recruit larger, more representative samples, potentially through extended data collection, varied recruitment strategies, or multi-site collaboration, to strengthen the reliability and generalisability of the findings to a wider student population. Considering the importance of year of study in the present study, further research should explore interactions between the year of study and social eating habits/practices. Additionally, qualitative studies could explore students’ lived experiences of loneliness and social eating habits and practices to gather more nuanced insights often missed by quantitative surveys. The previous qualitative study in the UK was influenced by the COVID-19 pandemic [19].

## 5. Conclusions

This study found relatively high levels of loneliness in the university student sample and identified significant associations with social eating habits and practices. While loneliness did not differ across most demographic groups, first-year undergraduate students reported significantly higher levels, suggesting the transition to university is a vulnerable period. Students who ate meals alone, including lunch, dinner, and snacks, reported significantly higher levels of loneliness. Furthermore, students who lacked mealtime companions or felt embarrassment when eating alone experienced greater loneliness. These findings support the development of targeted initiatives to facilitate social interactions around shared mealtimes at university. Future research should examine the long-term relationship between social eating practices and loneliness and explore these dynamics across more diverse student populations to inform scalable and effective interventions.

## Figures and Tables

**Table 1:** Demographic characteristics of survey respondents.

Participant Characteristic	N (%)
Age ^1^	
<18 years	1 (0.4%)
18–20 years	127 (49.8%)
21–23 years	67 (26.3%)
24–26 years	20 (7.8%)
27–29 years	20 (7.8%)
30 years	20 (7.8%)
Gender ^2^	
Male	52 (20.6%)
Female	191 (75.8%)
Other	9 (3.6%)
Ethnicity ^3^	
White	154 (61.8%)
Mixed/Multiple ethnic groups	8 (3.2%)
Asian/Asian British	76 (30.5%)
Black/African/Caribbean/Black British	6 (2.4%)
Any other ethnic group	5 (2.0%)
Country of origin ^4^	
UK	173 (70.9%)
Outside of the UK	71 (29.1%)
Length living in the UK ^5^	
Less than one year	30 (42.9%)
Between 1–3 years	15 (21.4%)
Between 4–5 years	5 (7.1%)
Over five years	20 (28.6%)
Religion ^6^	
Christian	63 (26.1%)
Buddhist	6 (2.5%)
Hindu	18 (7.5%)
Muslim	15 (6.2%)
Sikh	2 (0.8%)
No religion	136 (56.4%)
Other	1 (0.4%)
Current living situation ^7^	
Living at home with parents or guardian	16 (6.3%)
Living in student halls of residence	88 (34.8%)
Living with other students in private accommodation	101 (39.9%)
Living alone in private accommodation	23 (9.1%)
Living with partner	18 (7.1%)
Living with partner and children	2 (0.8%)
Other	5 (2.0%)
Number of people living in the household ^8^	
2	16 (7.7%)
3	22 (10.6%)
4	40 (19.3%)
5	19 (9.2%)
>5 people	110 (53.1%)
Year of study ^9^	
Undergraduate 1st year	68 (27.1%)
Undergraduate 2nd year	49 (19.5%)
Undergraduate 3rd year	45 (17.9%)
Undergraduate 4th year	10 (4.0%)
Postgraduate Masters (any year)	35 (13.9%)
Postgraduate PhD (any year)	35 (13.9%)
Other	9 (3.6%)
University Faculty ^10^	
Faculty of Arts	51 (21.3%)
Faculty of Science, Engineering and Medicine	132 (55.0%)
Faculty of Social Sciences	57 (23.8%)

^1^ N = 255, ^2^ N = 252, ^3^ N = 249, ^4^ N = 244, ^5^ N = 70, ^6^ N = 241, ^7^ N = 253, ^8^ N = 207, ^9^ N = 251, ^10^ N = 240. Other gender includes participants who identified as non-binary or preferred to self-describe.

**Table 2:** Levels of loneliness amongst participants.

Variable	Mean (SD)/N (%)
Loneliness (ULS-8) total score	18.27 (4.897)
Loneliness (Categories)	
No severe loneliness indicated	214 (83.9%)
Severe loneliness indicated	41 (16.1%)

**Table 3:** Loneliness related to types of meals eaten alone or with others and venues eaten at alone or with others.

Variables	Eating Alone (n) M (SD)	Eating with Others (n) M (SD)	Mean Diff.	*t*	*p*	Cohen’s d 95% CI
Meals eaten alone						
Breakfast	N = 183 18.55 (4.744)	N = 72 17.57 (5.235)	0.98	1.45	0.150	0.20 −0.07, 0.47
Morning snack	N = 122 18.98 (4.831)	N = 133 17.62 (4.884)	1.36	2.23	**0.026**	0.28 0.03, 0.53
Lunch	N = 142 19.06 (4.827)	N = 113 17.29 (4.827)	1.77	2.90	**0.004**	0.37 0.12, 0.61
Afternoon snack	N = 135 19.21 (4.692)	N = 120 17.23 (4.929)	1.98	3.29	**0.001**	0.41 0.16, 0.66
Dinner	N = 119 19.67 (4.728)	N = 136 17.05 (4.727)	2.62	4.42	**<0.001**	0.55 0.30, 0.81
Venues eaten at alone						
A university owned or other café on campus	N = 108 19.01 (5.127)	N = 147 17.73 (4.665)	1.28	2.07	**0.040**	0.26 0.01, 0.51
Outside on campus	N = 116 18.91 (4.814)	N = 139 17.74 (4.919)	1.17	1.91	0.057	0.24 −0.00, 0.48
At home	N = 184 18.91 (4.755)	N = 71 16.63 (4.911)	2.28	3.39	**<0.001**	0.47 0.20, 0.75
An eating venue off campus	N = 40 20.18 (4.236)	N = 215 17.92 (4.939)	2.26	2.71	**0.007**	0.47 0.13, 0.81

*p* values in bold indicate significance < 0.05.

**Table 4:** Loneliness differences by reasons for eating alone, feelings experienced and digital technology use.

Variables	Comparison	Direction	Mean Diff. (SE)	*p*	Test Statistic
Reasons for eating alone	Do not eat alone				F (10, 242) = 3.019, *p* = 0.001, η^2^ = 0.11, CI: 0.02, 0.15
Apprehensive about reaching out		Higher loneliness	6.675 (1.901)	**0.022**	
Apprehensive about eating with others		Higher loneliness	6.075 (1.581)	**0.007**	
No one to eat with		Higher loneliness	5.042 (1.327)	**0.008**	
Feelings experienced when eating alone					F (7, 246) = 4.834, *p* < 0.001, η^2^ = 0.12, CI: 0.04, 0.18
	Embarrassed vs. happy	Lower loneliness in happy group	−3.927 (1.259)	**0.042**	
	Carefree vs. lonely	Lower loneliness in carefree group	−3.562, (0.893)	**0.002**	
	Carefree vs. embarrassed	Lower loneliness in carefree group	−4.173 (1.049)	**0.002**	
Feelings experienced when eating with others					F (6, 245) = 6.320, *p* < 0.001, η^2^ = 0.13, CI: 0.05, 0.20
	Happy vs. uncomfortable	Lower loneliness in happy group	−2.596 (0.796)	**0.021**	
	Happy vs. embarrassed	Lower loneliness in happy group	−5.868 (1.475)	**0.002**	
	Carefree vs. uncomfortable	Lower loneliness in carefree group	−3.303 (0.847)	**0.002**	
	Carefree vs. embarrassed	Lower loneliness in carefree group	−6.576 (1.503)	**<0.001**	
	Carefree vs. none	Lower loneliness in carefree group	−3.433 (1.118)	**0.038**	
Small electronic device use					F (4, 250) = 3.955, *p* = 0.004, η^2^ = 0.06, CI: 0.01, 0.11
	Always vs. often	Higher loneliness in always group	2.167 (0.750)	**0.034**	
	Always vs. sometimes	Higher loneliness in always group	2.623 (0.879)	**0.026**	
	Always vs. rarely	Higher loneliness in always group	=3.784 (1.340)	**0.041**	
Watching TV during mealtimes					F (4, 249) = 3.040, *p* = 0.018, η^2^ = 0.05, CI: 0.00, 0.09
	Always vs. sometimes	Higher loneliness in always group	4.395 (1.336)	**0.010**	

*p* values in bold indicate significance < 0.05.

## Data Availability

The data presented in this study are available on request from the corresponding author due to privacy restrictions.

## Supplementary Materials

The following supporting information can be downloaded at: https://www.mdpi.com/article/10.3390/ijerph23060739/s1, social eating practices, questions, and response options, and coding and scoring of social eating practices questions.
