# Supplementary material for: Eating Together, Eating Alone: A Cross-Sectional Survey of Associations Between Social Eating Contexts, Mealtime Emotions, Technology Use, and Loneliness in UK University Students
Source: Int J Environ Res Public Health. 2026 Jun 1;23(6):739. doi: 10.3390/ijerph23060739 (PMC13299427; doi:10.3390/ijerph23060739)
Supplement: Supplementary file 1 [file ijerph-23-00739-s001.zip › ijerph-4227719-supplementary.pdf]

Eating together, eating alone: A cross-sectional survey of associations between social eating contexts, mealtime emotions, technology use, and loneliness in UK university students - Supplementary Information

Social eating habits questions and response options

Section 1 guidance Section 1: These questions are related to your social eating habits at university. We would like you to think about your social eating habits **during the current term** on a **typical day**.

Q1 Please select all options that apply to you

	Breakfast (6am-9am) (1)	Mid-morning snack (10am-11am) (2)	Lunch (12pm-2pm) (3)	Afternoon snack (3pm-4pm) (4)	Dinner (5pm-8pm) (5)	Prefer not to say (6)
On a typical day I usually eat (1)	☐	☐	☐	☐	☐	☐
On a typical day I usually skip (2)	☐	☐	☐	☐	☐	☐

Q2 Please select all options that apply to you

	Breakfast (6am-9am) (1)	Mid-morning snack (10am-11am) (2)	Lunch (12pm-2pm) (3)	Afternoon snack (3pm-4pm) (4)	Dinner (5pm-8pm) (5)	Prefer not to say (6)
On a typical day I usually eat alone during these mealtimes (1)	☐	☐	☐	☐	☐	☐
On a typical day I usually eat with others during these mealtimes (2)	☐	☐	☐	☐	☐	☐

Q3 Please select all options that apply to you

	A university owned eating venue on-campus (1)	Another café on-campus (e.g., Café Nero, Pret) (2)	Outside on-campus (e.g. in green space) (3)	Mobile / pop-up food truck on-campus (4)	At home (incl. university halls of residence) (5)	An eating venue off-campus (6)	Other (7)	All the above (8)	Prefer not to say (9)
I usually eat alone in these places (1)	▢	▢	▢	▢	▢	▢	▢	▢	▢
I usually eat with others in these places (2)	▢	▢	▢	▢	▢	▢	▢	▢	▢

Q4 I eat alone because (please select the one option that applies to you the most)

- ○ N/A (I don't eat alone) (1)
- ○ I am apprehensive about reaching out to connect with others (2)
- ○ I am apprehensive about eating with others (3)
- ○ I prefer not to communicate with others during mealtimes (4)
- ○ I don’t have anyone to eat with during mealtimes (5)
- ○ I prefer to be with my own thoughts (6)
- ○ I prefer to read a book (7)
- ○ I prefer to study during mealtimes (8)

○ I prefer using my phone (e.g., browsing news, browse social media, watching videos, watching TV show) (9)

○ I prefer using my phone to connect with others virtually (10)

○ Other (please specify) (11) ____

○ Prefer not to say (12)

Q5 For the purpose of meeting **new acquaintances**, I would typically choose to eat at (please select the one option that applies to you the most)

○ A university owned eating venue on-campus (1)

○ Another café on-campus (e.g., Café Nero, Pret) (2)

○ Outside on-campus (e.g. in green space) (3)

○ Mobile / pop-up food truck on-campus (4)

○ At home (incl. university halls of residence) (5)

○ An eating venue off-campus (6)

○ Other (please specify) (7) ____

○ All the above (8)

○ I would prefer not to meet in an eating venue (9)

○ Prefer not to say (10)

Q6a When socialising with **new acquaintances** I would typically choose to have a (please select the one option that applies to you the most)

- ◯ Snack (1)
- ◯ Drink (2)
- ◯ Confectionary (3)
- ◯ Light meal (e.g. sandwich) (4)
- ◯ Full meal (5)
- ◯ Takeaway (6)
- ◯ Home-made meal (7)
- ◯ Other (please specify) (8) ____
- ◯ Prefer not to say (9)

Q6b When socialising with **close friends** I would typically choose to have a (please select the one option that applies to you the most)

- ◯ Snack (1)
- ◯ Drink (2)
- ◯ Confectionary (3)
- ◯ Light meal (e.g. sandwich) (4)
- ◯ Full meal (5)
- ◯ Takeaway (6)
- ◯ Home-made meal (7)
- ◯ Other (please specify) (8) ____
- ◯ Prefer not to say (9)

7a When I **eat alone** at a university venue, I feel (please select the one option that applies to you the most)

- Happy / enjoying myself (1)
- Carefree / relaxed (2)
- Uncomfortable / disturbed (3)
- Lonely (4)
- Sad (5)
- Embarrassed / ashamed (6)
- Other (please specify) (7) ____
- None of these (8)
- Prefer not to say (9)

Q7b When I **eat with other students (not close friends)** at a university venue, I feel (please select the one option that applies to you the most)

- Happy / enjoying myself (1)
- Carefree / relaxed (2)
- Uncomfortable / disturbed (3)
- Lonely (4)
- Sad (5)
- Embarrassed / ashamed (6)
- Other (please specify) (7) ____
- None of these (8)
- Prefer not to say (9)

Q8 During mealtimes I usually

	Never (1)	Rarely (2)	Sometimes (3)	Often (4)	Always (5)	Prefer not to say (6)
Use small electronic devices (e.g., smartphone, tablet, laptop) (1)	◯	◯	◯	◯	◯	◯
Watch TV (2)	◯	◯	◯	◯	◯	◯

Q9 During mealtimes I like using digital technologies to connect with others

- ◯ Yes (3)
- ◯ No (5)
- ◯ Prefer not to say (4)

Q10 During mealtimes I use the following platforms

- ◯ Subscription service (e.g., Netflix, Prime videos, Disney+) (1)
- ◯ YouTube (2)
- ◯ Social networking apps (e.g., WhatsApp, Messenger) (3)
- ◯ News websites (4)
- ◯ Moodle (5)
- ◯ Other (please specify) (6) ____
- ◯ Prefer not to say (7)

Q11

Are you aware of any apps available that aim to **support socialising during mealtimes**?

- ○ Yes (3)
- ○ No (5)
- ○ Prefer not to say (4)

Coding and scoring of social eating habits questions

Variable Name	Variable Label	Coding Instructions	Measurement Scale	Additional Information
SEH_Q1a_break	On a typical day I usually eat breakfast	1 = Selected option, 2 = Did not select option	Nominal	For Q1-Q3, participants were asked to select all options that apply
SEH_Q1a_msnack	On a typical day I usually eat morning snack	1 = Selected option, 2 = Did not select option	Nominal	
SEH_Q1a_lunch	On a typical day I usually eat lunch	1 = Selected option, 2 = Did not select option	Nominal	
SEH_Q1a_asnack	On a typical day I usually eat afternoon snack	1 = Selected option, 2 = Did not select option	Nominal	
SEH_Q1a_dinn	On a typical day I usually eat dinner	1 = Selected option, 2 = Did not select option	Nominal	
SEH_Q1a_pnts	On a typical day I usually eat - prefer not to say	99 = prefer not to say, 2 = Did not select option	Nominal	

SEH_Q1b_break	On a typical day I usually skip breakfast	1 = Selected option, 2 = Did not select option	Nominal	
SEH_Q1b_msnack	On a typical day I usually skip morning snack	1 = Selected option, 2 = Did not select option	Nominal	
SEH_Q1b_lunch	On a typical day I usually skip lunch	1 = Selected option, 2 = Did not select option	Nominal	
SEH_Q1b_asnack	On a typical day I usually skip afternoon snack	1 = Selected option, 2 = Did not select option	Nominal	
SEH_Q1b_dinn	On a typical day I usually skip dinner	1 = Selected option, 2 = Did not select option	Nominal	
SEH_Q1b_pnts	On a typical day I usually skip - prefer not to say	99 = prefer not to say, 2 = Did not select option	Nominal	
SEH_Q2a_break	On a typical day I usually eat alone during breakfast	1 = Selected option, 2 = Did not select option	Nominal	
SEH_Q2a_msnack	On a typical day I usually eat alone during morning snack	1 = Selected option, 2 = Did not select option	Nominal	

SEH_Q2a_lunch	On a typical day I usually eat alone during lunch	1 = Selected option, 2 = Did not select option	Nominal	
SEH_Q2a_asnack	On a typical day I usually eat alone during afternoon snack	1 = Selected option, 2 = Did not select option	Nominal	
SEH_Q2a_dinn	On a typical day I usually eat alone during dinner	1 = Selected option, 2 = Did not select option	Nominal	
SEH_Q2a_pnts	On a typical day I usually eat alone - prefer not to say	99 = prefer not to say, 2 = Did not select option	Nominal	
SEH_Q2b_break	On a typical day I usually eat with others during breakfast	1 = Selected option, 2 = Did not select option	Nominal	
SEH_Q2b_msnack	On a typical day I usually eat with others during morning snack	1 = Selected option, 2 = Did not select option	Nominal	
SEH_Q2b_lunch	On a typical day I usually eat with others during lunch	1 = Selected option, 2 = Did not select option	Nominal	
SEH_Q2b_asnack	On a typical day I usually eat with others during afternoon snack	1 = Selected option, 2 = Did not select option	Nominal	

SEH_Q2b_dinn	On a typical day I usually eat with others during dinner	1 = Selected option, 2 = Did not select option	Nominal	
SEH_Q2b_pnts	On a typical day I usually eat with others - prefer not to say	99 = prefer not to say, 2 = Did not select option	Nominal	
SEH_Q3a_univenue	I usually eat alone in a university owned eating venue on-campus	1 = Selected option, 2 = Did not select option	Nominal	
SEH_Q3a_campuscafe	I usually eat alone in another café on-campus	1 = Selected option, 2 = Did not select option	Nominal	
EatAlone_Campus	condensed eat alone uni campus venues	1 = Selected option, 2 = Did not select option	Nominal	
SEH_Q3a_campusout	I usually eat alone outside on-campus	1 = Selected option, 2 = Did not select option	Nominal	
SEH_Q3a_campuspop	I usually eat alone at a mobile / pop-up food truck on-campus	1 = Selected option, 2 = Did not select option	Nominal	
EatAlone_CampusOutside	condensed eat alone uni campus outside	1 = Selected option, 2 = Did not select option	Nominal	

SEH_Q3a_home	I usually eat alone at home (incl. university halls of residence)	1 = Selected option, 2 = Did not select option	Nominal	
SEH_Q3a_offcampus	I usually eat alone at an eating venue off-campus	1 = Selected option, 2 = Did not select option	Nominal	
SEH_Q3a_other	I usually eat alone - other	1 = Selected option, 2 = Did not select option	Nominal	
SEH_Q3a_allabove	I usually eat alone - all of the above	1 = Selected option, 2 = Did not select option	Nominal	
SEH_Q3a_pnts	I usually eat alone - pnts	99 = prefer not to say, 2 = Did not select option	Nominal	
SEH_Q3b_univenue	I usually eat with others in a university owned eating venue on-campus	1 = Selected option, 2 = Did not select option	Nominal	
SEH_Q3b_campuscafe	I usually eat with others in another café on-campus	1 = Selected option, 2 = Did not select option	Nominal	
EatWithOthers_Campus	condensed eat with others uni campus venues	1 = Selected option, 2 = Did not select option	Nominal	

SEH_Q3b_campusout	I usually eat with others outside on-campus	1 = Selected option, 2 = Did not select option	Nominal	
SEH_Q3b_campuspop	I usually eat with others at a mobile / pop-up food truck on-campus	1 = Selected option, 2 = Did not select option	Nominal	
EatWithOthers_CampusOutside	condensed eat with others uni campus outside	1 = Selected option, 2 = Did not select option	Nominal	
SEH_Q3b_home	I usually eat with others at home (incl. university halls of residence)	1 = Selected option, 2 = Did not select option	Nominal	
SEH_Q3b_offcampus	I usually eat with others at an eating venue off-campus	1 = Selected option, 2 = Did not select option	Nominal	
SEH_Q3b_other	I usually eat with others - other	1 = Selected option, 2 = Did not select option	Nominal	
SEH_Q3b_allabove	I usually eat with others - all of the above	1 = Selected option, 2 = Did not select option	Nominal	
SEH_Q3b_pnts	I usually eat with others - pnts	99 = prefer not to say, 2 = Did not select option	Nominal	

SEH_Q4	Main reason for eating alone	1 = N/A (I don't eat alone), 2 = I am apprehensive about reaching out to connect with others, 3 = I am apprehensive about eating with others, 4 = I prefer not to communicate with others during mealtimes, 5 = I don't have anyone to eat with during mealtimes, 6 = I prefer to be with my own thoughts, 7 = I prefer to read a book, 8 = I prefer to study during mealtimes, 9 = I prefer using my phone (e.g., browsing news, browse social media, watching videos, watching TV show), 10 = I prefer using my phone to connect with others virtually, 11 = Other (please specify), 99 = prefer not to say	Nominal	
SEH_Q4_condensed	Main reason for eating alone condensed	1 = N/A (I don't eat alone), 2 = I am apprehensive about reaching out or eating with or don't have anyone to eat with, 3 = I prefer not to communicate with others and do something else e.g., be with my own thoughts, read a book, study, 4 = I prefer using my phone (e.g., browsing news, browse social media, watching videos, watching TV show), 5 = I prefer using my phone to connect with others virtually, 6 =	Nominal	

		Other (please specify), 99 = prefer not to say		
SEH_Q4_11_text	Specify reason for eating alone	Text response	N/A - String variable	
SEH_Q5	Eating location when meeting new acquaintances	1 = A university owned eating venue on-campus, 2 = Another café on-campus (e.g., Café Nero, Pret), 3 = Outside on-campus (e.g. in green space), 4 = Mobile / pop-up food truck on-campus, 5 = At home (incl. university halls of residence), 6 = An eating venue off-campus, 7 = Other (please specify), 8 = All the above, 9 =I would prefer not to meet in an eating venue, 99 = prefer not to say	Nominal	
SEH_Q5_condensed	Eating location when meeting new acquaintances condensed	1 = An eating venue on-campus (uni owned or other Cafe), 2 = Outside on-campus (e.g. in green space or pop-up truck), 3 = At home (incl. university halls of residence), 4 = An eating venue off-campus, 5 = Other (please specify), 6 = All the above, 7 = I would prefer not to meet in an eating venue, 99 = prefer not to say	Nominal	

SEH_Q5_7_text	Specify other location new acquaintances	Text response	N/A - String variable	
SEH_Q6a	Consume which food with new acquaintances	1 = Snack, 2 = Drink, 3 = Confectionary, 4 = Light meal (e.g. sandwich), 5 = Full meal, 6 = Takeaway, 7 = Home-made meal, 8 = Other (please specify), 99 = prefer not to say	Nominal	
SEH_Q6a_8_text	Specify other food	Text response	N/A - String variable	
SEH_Q6b	Consume which food with close friends	1 = Snack, 2 = Drink, 3 = Confectionary, 4 = Light meal (e.g. sandwich), 5 = Full meal, 6 = Takeaway, 7 = Home-made meal, 8 = Other (please specify), 99 = prefer not to say	Nominal	
SEH_Q6b_8_text	Specify other food	Text response	N/A - String variable	
SEH_Q7a	Feelings eating alone at uni venue	1 = Happy / enjoying myself, 2 = Carefree / relaxed, 3 = Uncomfortable / disturbed, 4 = Lonely, 5 = Sad, 6 = Embarrassed / ashamed, 7 = Other (please specify), 8 = None of these, 99 = prefer not to say	Nominal	

SEH_Q7a_7_text	Specify other feeling	Text response	N/A - String variable	
SEH_Q7b	Feelings eating with other students (not close friends) at uni venue	1 = Happy / enjoying myself, 2 = Carefree / relaxed, 3 = Uncomfortable / disturbed, 4 = Lonely, 5 = Sad, 6 = Embarrassed / ashamed, 7 = Other (please specify), 8 = None of these, 99 = prefer not to say	Nominal	
SEH_Q7b_7_text	Specify other feeling	Text response	N/A - String variable	
SEH_Q8a	Use of small electronic devices (e.g., smartphone, tablet, laptop) during mealtimes	1 = Never, 2 = Rarely, 3 = Sometimes, 4 = Often, 5 = Always, 99 = prefer not to say	Ordinal	
SEH_Q8a_ condensed	Use of small electronic devices (e.g., smartphone, tablet, laptop) during mealtimes condensed	1 = Never or Rarely, 2 Sometimes, 3 = Often or Always, 99 = prefer not to say	Ordinal	
SEH_Q8b	Watching TV during mealtimes	1 = Never, 2 = Rarely, 3 = Sometimes, 4 = Often, 5 = Always, 99 = prefer not to say	Ordinal	
SEH_Q8b_ condensed	Watching TV during mealtimes condensed	1 = Never or Rarely, 2 Sometimes, 3 = Often or Always, 99 = prefer not to say	Ordinal	

SEH_Q9	Like use of digital technologies to connect with others during mealtimes	1 = Yes, 2 = No, 99 = prefer not to say	Nominal	
SEH_10	Use of platforms during mealtimes	1 = Subscription service (e.g., Netflix, Prime videos, Disney+), 2 = YouTube, 3 = Social networking apps (e.g., WhatsApp, Messenger), 4 = News websites, 5 = Moodle, 6 = Other (please specify), 99 = prefer not to say	Nominal	
SEH_Q10_6_text	Specify other platform	Text response	N/A - String variable	
SEH_Q11	Awareness of apps that support socialising during mealtimes	1 = Yes, 2 = No, 99 = prefer not to say	Nominal	
